# Monthly Summary

**Published:** 1897-09

**Authors:** 


### Monthly Summary.

PITIABLE CONDITION OF A GIRL FROM USE OF X-RAYS,—Miss Josie MacDonald of 9 West Forty-fifth street, New York, is lying at her home in a pitiable condition as a result of a 20-minute exposure to X-rays during the latter part of June, when an X-ray photograph of her upper left jaw was taken by a man whose name is supposed to be O'Connor. The young woman had for some time previous been suffering with intense pains in her jaw. She went to Nelson Shields and George F. Jernigan, dentists, who diagnosed her disease as necrosis.

In order to determine the exact cause they decided to have an X-ray photograph taken of the jaw to see if there was an old root there. The young woman was taken to the laboratory of Mr. O'Connor, who had been recommended to the dentists by Dr. Lee. The first photograph taken was not a success on account of too short an exposure, and it was resolved to have another one taken. The second photograph was taken the next day, with an exposure of 20 minutes, and proved the dentists' diagnosis correct.

A few days afterward Miss MacDonald's face began to swell and grew black. Dr. Henry Griswold was called, and he commenced treating the case as he would an ordinary burn. The hair on one side of the patient's head began to fall out, and in a short time that part of her scalp was bare. Her face was blistered, and sores formed all the way down her neck, shoulders and arm. A scab formed over the blister, and when it fell off it disclosed the raw flesh. Her left ear swelled up, and she lost her hearing on that side. Constant applications of oil have been necessary day and night to allay the pain arising from exposure of the flesh to the air.

Dr. Griswold said that he could not tell who was responsible for the condition of his patient.

Miss MacDonald has placed her case in the hands of Sullivan & Cromwell, attorneys, at 45 Wall street, and an action will shortly be commenced against the dentists.

---

FILLING FRAIL TEETH.—We should remember when dealing with frail teeth, having large cavaties, that great masses of gold placed in contact with frail enamel walls must result in disaster from the mere effects of expansion caused by extremes of heat and cold. At one moment we drink ice water, bringing the temperature of the teeth and gold fillings down to thirty, and then we deluge the mouth with scalding hot tea, the result being a rapid and unequal rise of temperature, the gold responding and expanding to a greater degree than the enamel.—Dr. Hill, *Items*.

---

THE BACTERIOLOGY OF BALDNESS.—The microbe which causes baldness has been discovered after patient research by Sabouraud, who by using cultures of the microbe has produced alopecia in the sheep, the guinea-pig and the rabbit. If, as has been often said, pyorrhea alveolaris is to the teeth what baldness is to the hair, we may expect shortly to have the pyorrhea microbe discovered, and perhaps then we may discover some specific treatment for this disorder, which seems to be much on the increase.

ROOT PERFORATION—A NEW METHOD OF TREATMENT—My first case of traumatic perforation occurred about six years ago, in a right upper lateral incisor, which I was called upon to crown. The root has been filled some years before with gutta percha. This I drilled out to what I judged to be about three-fourths the depth of the root, which at this point seemed to take a bend to the left. Having reamed out the canal, and fearing it was not quite deep enough to accommodate the pin. I unwisely proceeded to drill the canal a little deeper. I had almost succeeded to my satisfaction, when the patient showed sudden signs of pain, and on withdrawing the drill, wiping out the canal, I found traces of blood. I immediately dried out the canal and carefully dressed it with oil of cloves. Next day the case showed a distinct swelling on the gum over the seat of the perforation, but within three days this swelling had entirely disappeared and all irritation had subsided. At a subsequent appointment I removed the dressing, dried the canal out thoroughly, and proceeded to seal the perforation in the following manner.

Having mixed copper amalgam quite thin I gently carried a small portion of it to the apex with a blunt-pointed instrument rounded at the end—in fact, an old excavator with the point broken off. The amalgam was gently spread round the upper portion of the canal, a rotary motion being used to carry it evenly around the interior. No pain was caused by this proceeding, but the patient was just aware of a very slight sensation while it was being done; this sensation subsided when the point was withdrawn. The interior of the cavity being thus lined, its depth was not seriously lessened. The patient was then dismissed till the following day, when the amalgam had hardened thoroughly, and at the next sitting a Richmond crown was set, and has done good service up to the present time.—John Girdwood, in *International Journal.*

---

TO OBTAIN absolute cleanliness of tooth before adjusting the rubber dam or for crown or bridge work, wipe off the tooth with extracts of eucalyptus or chloroform.—*Dental News.*

THE FOLLOWING RULES for the care of the eyes conform to well-established laws of eye-physiology :

Avoid reading and study by poor light.

Light should come from the side, and not from the back or from the front.

Do not read or study while suffering great bodily fatigue or during recovery from illness.

Do not read while lying down.

Do not use the eyes too long at a time for near work, but give them occasional periods of rest.

Reading and study should be done systematically.

During study avoid the stooping position, or whatever tends to produce congestion of the head and face.

Select well-printed books.

Correct errors of reflection with proper glasses.

Avoid bad hygienic conditions and the use of alcohol and tobacco.

Take sufficient exercise in the open air.

Let the physical keep pace with the mental culture, for asthenopia is most usually observed in those who are lacking in physical development,—*Pacific Med. Record.*

---

DIFFICULT PARTIAL IMPRESSIONS.—Several weeks ago a lady presented, desiring a partial upper denture. An examination of the oral cavity revealed a V-shaped arch with several sound teeth, posteriorly and anterioriy. Wherever possible I prefer taking plaster impressions in partial cases; I, therefore, proceeded in the usual manner to get a plaster impression. The teeth were at unfavorable angles, seriously interfering with the work. After several unsuccessful attempts and spending considerable time in trying to patch together broken pieces which were more intricate than a Chinese puzzle, I hit upon the following method: That portion of the plaster which came out intact, viz , the palatal surface, festooning teeth lingually, and partially covering the ridge where teeth were absent, I replaced carefully in the impression-tray, flowing *very* soft plaster where the first impression had been imperfect. I next passed the tray and plaster quickly into the arch, retaining it in position until the new plaster had set in. The cup was then easily removed, the new plaster fractured cleanly and in large pieces, some, however, remaining cemented to the original impression, making it a simple task to reunite the whole. The result was a perfect model of all the teeth, ridge, palatal surface, and condyles, from which I made a well-fitting plate.—Homer Heberling, D. D. S., in *Items of Interest.*

---

SUICIDE BY TAKING COCAIN.—An inquest was held on November 19th, on the body of a woman aged twenty-two, the wife of a wealthy American gentleman. From the evidence it appears that the deceased had suffered very much from toothache and neuralgia, for which the medical man had prescribed quinine and other remedies. On the 14th she was taking tea with her doctor when she suddenly complained of intense toothache. He took her to his surgery and placed some crystals of cocain in her tooth, putting back the cocain into the drawer he had taken it from. He did not see her again till he was sent for to the Hotel Cecil, where he was horrified to find she had abstracted the cocain bottle, and had taken its contents, some fifty grains, causing death. The jury, after a short deliberation, returned a verdict of suicide during a fit of temporary insanity. We can not understand how a person possessed of wealth should have allowed her teeth to become a source of such continual torture as appears evident. A caution also is needed when dealing with patients, to see that dangerous drugs are placed securely out of their way.—*British Journal.*

---

COCAINE solution decomposes in forty-eight hour. You need to prepare it fresh when you use it. If it is more than forty-eight hours old it will not give good results.—Dr. Meeker in *International.*

BLOOD-POISONING AFTER TOOTH EXTRACTION.—Dr. Port, of Munich, remarks that, "when we consider the large quantity of micro-organisms which flourish in the mouth, it is extraordinary that dental extractions are not more frequently a source of infection." Dr. Miller's book cites only sixty cases, of which about half the number terminated fatally, while the other half recovered sooner or later. Death generally occurred from septicemia, pyemia or meningitis. He gives a recent case of a young and vigorous man whose lower molar had been extracted by means of the key. He developed fever and died in four days. The autopsy revealed a large abscess in the neck, the pleural cavities held a large quantity of fetid brown pus, while the pericardium also contained pus. The abscess disclosed streptococci and diplococci, and the latter resembled the salivary septicemic microbe described by Miller.—*Brit. Jour. of Dent. Science.*

---

NEVERMIND THE OTHER FELLOW.—As a rule I do not like to have a patient tell me where he has been before, when he comes into my office. I do not wish to know who has done this or that. I give such patients very little encouragement to talk, and as a rule they keep things to themselves.—G. Newkirk, in *Review.*

---

INSTEAD of the method so frequently employed of heating gutta percha held in the plyers over the alcohol flame, use a disc or slab of porcelain; place it in contact with the heat of the flame; put your pellets of gutta-percha on this, and when sufficiently soft to be kneaded, work with cold instruments into the cavity.—*Dental News.*

---

A CURE FOR HICCOUGH.—A never-failing cure for hiccough that gives prompt relief is to draw in as much air as the lungs will hold, and retain it as long as possible. Once is generally sufficient, but if necessary it may be repeated.—*Dental News.*

